# Supplementary material for: A cross sectional study to examine factors influencing COVID-19 vaccine acceptance, hesitancy and refusal in urban and rural settings in Tamil Nadu, India
Source: PLoS One. 2022 Jun 9;17(6):e0269299. doi: 10.1371/journal.pone.0269299 (PMC9182563; doi:10.1371/journal.pone.0269299)
Supplement: S2 Appendix — (PDF) [file pone.0269299.s002.pdf]

Informed Consent Form

I ____ agree to take part in the study, conducted by Prof. Dr. Surapaneni Krishna Mohan, Panimalar Medical College Hospital & Research Institute, Varadharajapuram, Poonamallee ,Chennai, 600123.

Title of the research project: COVID-19 **V**accine **IN**DIA **C**ommunication**, A**cceptance**, P**ractice (CO-VIN-CAP). The study bearing protocol number: PMCHRI-IHEC-029 has been approved from Panimalar Medical College Hospital & Research Institute-Institutional Human Ethics Committee (PMCHRI-IHEC): CDSCO Registration No. ECR/1399/Inst/TN/2020. Approval No: PMCH&RI/IHEC/2021/037 dated 13.01.2021.

I acknowledge that I have read the information and the same has been explained to me clearly by the Principal Investigator.

I know about -
- Possible risks in the study
- Benefits of the study
- Compensation if any and if applicable
- Confidentiality of all the information
- Withdrawal from the study at any stage
- If more information is required, whom to contact
- Complaints regarding the study, whom to contact

I agree to give the necessary information and participate in the study.

Signature of the participant/patient

Thumb impression

Legally accepted representative (In case the participant is illiterate)

Address:

Date:

தகவலறிந்த ஒப்புதல் படிவம்

திரு/ திருமதி/ செல்வி ____எனும் நான், பேராசிரியர் Dr.சூரப்பனேணி கிருஷ்ண மோகன், பனிமலர் மருத்துவ கல்லூரி மருத்துவமனை மற்றும் ஆராய்ச்சி நிறுவனம் நடத்தும் ஆய்வில் பங்கேற்க ஒப்புக்கொள்கிறேன்.

ஆராய்ச்சி திட்டத்தின் தலைப்பு: COVID - 19 தடுப்பூசி இந்திய தொடர்பு / ஏற்பு / பயிற்சி (CO - VIN - CAP). PMCHRI – IHEC - 029 என்ற நெறிமுறை எண் கொண்ட ஆய்வு, பனிமலர் மருத்துவ கல்லூரி மருத்துவமனை மற்றும் ஆராய்ச்சி நிறுவனத்தில் இயங்கும் - நிறுவன மனித நெறிமுறைகள் குழுவால் அங்கீகரிக்கப்பட்டது (PMCHRI - IHEC) : CDSCO பதிவு எண்: ECR/1399/INST/ TN/2020.
ஒப்புதல் எண் : PMCH & RI/IHEC/2021/037 தேதி : 13.01.2021.

நான் தகவலை படித்தேன் என்பதையும், முதன்மை புலனாய்வாளரால் எனக்கு தெளிவாக விளக்கப்பட்டது என்பதையும் ஒப்புக்கொள்கிறேன்.

இந்த ஆய்வின்,

சாத்தியமான அபாயங்கள்.
பலன்கள்.
இழப்பீடு (ஏதேனும் இருந்தால் மற்றும் பொருந்தினால்).
அனைத்து தகவல்களின் ரகசியத்தன்மை.
எந்நிலையிலும் விலகுதல்.
மேலும் தகவல் தேவைப்பட்டால், யாரை தொடர்புகொள்ள வேண்டும்.
புகார்கள் தொடர்பாக யாரை தொடர்புகொள்ள வேண்டும்.

என்பவை குறித்து நான் அறிவேன். ஆகையால் தேவையான தகவல்களை வழங்கவும் மற்றும் ஆய்வில் பங்கேற்கவும் ஒப்புக்கொள்கிறேன்.

பங்கேற்பாளர்/ நோயாளியின் கையொப்பம் :

கைரேகை பதிவு :

சட்டப்பூர்வமாக ஏற்றுக்கொள்ளப்பட்ட பிரதிநிதி (பங்கேற்பாளர் படிப்பறிவற்றவராக இருந்தால்)

முகவரி :

தேதி :
