# Supplementary material for: A cross sectional study to examine factors influencing COVID-19 vaccine acceptance, hesitancy and refusal in urban and rural settings in Tamil Nadu, India
Source: PLoS One. 2022 Jun 9;17(6):e0269299. doi: 10.1371/journal.pone.0269299 (PMC9182563; doi:10.1371/journal.pone.0269299)
Supplement: S4 Appendix — (DOCX) [file pone.0269299.s004.docx]

**S4Appendix: Health status of the respondents**

| **Variables** | **Attributes** | **Total n(%)** | **Urban, n (%)** | **Rural, n (%)** |
| --- | --- | --- | --- | --- |
| Do you have any underlying physician confirmed illnesses? (n=3080) | Yes | 702 (23) | 406 (21) | 292 (25) |
|  | No | 2240 (73) | 1397 (74) | 836 (71) |
|  | I do not know | 138 (4) | 88 (5) | 50 (4) |
| Enrolled in any health insurance program (n=3072) | No | 2127 (69) | 1292 (69) | 825 (70) |
|  | Private | 483 (16) | 303 (16) | 179 (15) |
|  | Government | 462 (15) | 290 (15) | 171 (15) |
| Have you tested for COVID-19? (n=3055) | Yes | 1667 (55) | 976 (52) | 682 (58) |
|  | No | 1163 (38) | 756 (40) | 405 (35) |
|  | Not willing to tell | 225 (7) | 139 (7) | 85 (7) |
| If yes, what was the result of your COVID-19 test? (n=1985) | Positive | 232 (12) | 148 (13) | 81 (10) |
|  | Negative | 1619 (82) | 907 (80) | 705 (83) |
|  | Not willing to tell | 134 (7) | 74 (7) | 59 (7) |
| If the COVID-19 test result was positive, how severe were your symptoms? (n=169) | No symptoms | 51 (30) | 35 (31) | 16 (29) |
|  | Mild Symptoms | 56 (33) | 38 (33) | 18 (33) |
|  | Moderate symptoms but did not seek help from a doctor | 33 (20) | 22 (19) | 11 (20) |
|  | Moderate symptoms and sought help from a doctor | 16 (9) | 11 (10) | 5 (9) |
|  | Severe symptoms and was hospitalized | 13 (8) | 8 (7) | 5 (9) |
| If the COVID-19 test result was positive, did you practice any home remedy to treat the infection? (n=158) | Yes | 71 (45) | 54 (50) | 17 (33) |
|  | No | 59 (37) | 36 (34) | 23 (45) |
|  | Not willing to tell | 28 (18) | 17 (16) | 11 (22) |
| If yes, please specify home remedy practiced? (n=33) | Natural herbs | 33 | 14 (100) | 16 (100) |
| Body mass index (BMI) (n=100) | <18.5 | 7 (2) | 4 (2) | 3 (4) |
|  | 18.5-22.9 | 3 (1) | 3 (1) | 0 |
|  | 23-24.9 | 9 (3) | 7 (3) | 2 (3) |
|  | >=25 | 295 (94) | 223 (94) | 69 (93) |
