# Supplementary material for: A cross sectional study to examine factors influencing COVID-19 vaccine acceptance, hesitancy and refusal in urban and rural settings in Tamil Nadu, India
Source: PLoS One. 2022 Jun 9;17(6):e0269299. doi: 10.1371/journal.pone.0269299 (PMC9182563; doi:10.1371/journal.pone.0269299)
Supplement: S5 Appendix — (DOCX) [file pone.0269299.s005.docx]

**S5 Appendix Distribution of Health status against acceptance of COVID-19 vaccine**

| **Variables** | **Attributes** | **Vaccine Acceptance n (%)** | **Vaccine Hesitant n (%)** | **Vaccine Rejection (%)** | **Likelihood Ratio Chi-Square (p-value)** |
| --- | --- | --- | --- | --- | --- |
| Do you have any underlying physician confirmed illnesses? (n=3060) | Yes (n=695) | 363(52) | 97(14) | 235(34) | 4.729 (0.316) |
|  | No (n=2227) | 1184(53) | 388(17) | 655(29) |  |
|  | I do not know (n=138) | 64(46) | 18(13) | 56(41) |  |
| Enrolled in any health insurance program (n=3054) | No (n=2114) | 1044(49) | 417(20) | 653(31) | 4.06 (0.398) |
|  | Private (n=477) | 304(64) | 48(10) | 125(26) |  |
|  | Government (n=463) | 260(57) | 34(7) | 166(36) |  |
| Have you tested for COVID-19? (n=3035) | Yes (n=1657) | 1025(62) | 322(19) | 310(19) | - |
|  | No (n=1154) | 523(45) | 139(12) | 492(43) |  |
|  | Not willing to tell (n=224) | 65(29) | 39(17) | 120(54) |  |
| If yes, what was the result of your COVID-19 test? (n=1644) | Positive (n=198) | 128(65) | 40(20) | 30(15) | - |
|  | Negative (n=1409) | 867(62) | 274(19) | 268(19) |  |
|  | Not willing to tell (n=37) | 22(59) | 7(19) | 8(22) |  |
| If the COVID-19 test result was positive, how severe were your symptoms? (n=172) | No symptoms (n=51) | 28(55) | 16(31) | 7(14) | 8.381 (0.397) |
|  | Mild Symptoms (n=58) | 39(67) | 7(12) | 12(21) |  |
|  | Moderate symptoms but did not seek help from a doctor (n=34) | 25(74) | 3(9) | 6(18) |  |
|  | Moderate symptoms and seek help from a doctor (n=16) | 13(81) | 2(13) | 1(6) |  |
|  | Severe symptoms and was hospitalized (n=13) | 8(62) | 2(15) | 3(23) |  |
| If the COVID-19 test result was positive, did you practice any home remedy to treat the infection? (n=161) | Yes (n=73) | 53(73) | 14(19) | 6(8) | 4.743 (0.315) |
|  | No (n=60) | 40(67) | 10(17) | 10(17) |  |
|  | Not willing to tell (n=28) | 13(46) | 8(29) | 7(25) |  |

Significance taken at p < 0.05 (In Bold)
