# Supplementary material for: A cross sectional study to examine factors influencing COVID-19 vaccine acceptance, hesitancy and refusal in urban and rural settings in Tamil Nadu, India
Source: PLoS One. 2022 Jun 9;17(6):e0269299. doi: 10.1371/journal.pone.0269299 (PMC9182563; doi:10.1371/journal.pone.0269299)
Supplement: Attachment — Submitted filename: Response_To_Reviewers.docx [file pone.0269299.s006.docx]

**Response to Reviewers**

**ACADEMIC EDITOR:** As the study has employed a big sample size and it has been found that the tables and the sample (n) does not match. Kindly clarify on similar terms. Also, provide ethical clearance no from the institution in the revised manuscript. Such issues would be dealt seriously in the revised version.

**Response:** The data had missing variables; therefore, all the questions were analysed separately. The Institutional ethical clearance was approved from the Panimalar Medical College Hospital & Research Institute-Institutional Human Ethics Committee (PMCHRI-IHEC): CDSCO Registration No. ECR/1399/Inst/TN/2020 with approval Number PMCH&RI/IHEC/2021/037 dated 13.01.2021.The same is mentioned in the manuscript.

**Journal Requirements**

1. Please ensure that your manuscript meets PLOS ONE's style requirements, including those for file naming.
**Response:** The manuscript is revised to ensure that it meets the PLOS ONE's style requirements.

2. We suggest you thoroughly copyedit your manuscript for language usage, spelling, and grammar. The name of the colleague or the details of the professional service that edited your manuscript. A copy of your manuscript showing your changes by either highlighting them or using track changes (uploaded as a *supporting information* file). A clean copy of the edited manuscript (uploaded as the new *manuscript* file)

**Response:** The manuscript is thoroughly copyedited for language usage, spelling, and grammar. The copy of your manuscript showing changes and the clean copy of the edited manuscript is uploaded as per the required name.

3. Please amend your current ethics statement to address the following concerns:
a) Did participants provide their written or verbal informed consent to participate in this study?

**Response:** The participants provided written informed consent to participate in the study. The informed consent sheet has been uploaded as a supporting material. The changes are made to the current ethics statement in the manuscript.

b) If consent was verbal, please explain i) why written consent was not obtained, ii) how you documented participant consent, and iii) whether the ethics committees/IRB approved this consent procedure.

**Response:** Not applicable, a written informed consent was obtained from the participants.

4. In your Data Availability statement, you have not specified where the minimal data set underlying the results described in your manuscript can be found. PLOS defines a study's minimal data set as the underlying data used to reach the conclusions drawn in the manuscript and any additional data required to replicate the reported study findings in their entirety. All PLOS journals require that the minimal data set be made fully available.

Upon re-submitting your revised manuscript, please upload your study’s minimal underlying data set as either Supporting Information files or to a stable, public repository and include the relevant URLs, DOIs, or accession numbers within your revised cover letter. Any potentially identifying patient information must be fully anonymized.

**Response:** We have created an excel sheet of the anonymized data set and provided the link where the anonymized data is stored. We have uploaded the data set as Supporting Information.

5. Please include captions for your Supporting Information files at the end of your manuscript, and update any in-text citations to match accordingly.

**Response:** The caption for the Supporting Information Files and in-text citation has been included and updated at the end of the manuscript.

**Reviewer #1:**

1. The study has been carried out in a rural area in Tamil Nadu. Shouldn't the corresponding author be from the institute where the data has been collected?

**Response:** The study has been carried out in both rural and urban settings. The corresponding author is responsible for the conceptualization of the study, study design, research questions and the institute in Tamil Nadu is responsible for the implementation of the study protocol and the data collection.

2. The study has been carried out in government PHC's across Tamil Nadu, however there has been no mention of permission being granted/ ethical clearance from the government to carry out this study. Kindly clarify.

**Response:** Yes, the study was carried out after permission was granted by the PHCs.

3. Were pregnant and lactating women included in the study? If they were, it would have contributed to more hesitancy. At this time, vaccines were still not recommended for pregnant or lactating women.

**Response:** We thank the reviewer for identifying the missing component in the exclusion criteria. The pregnant and lactating women were excluded from the study. The exclusion criteria have been revised accordingly.

4. The sample size is 3130. However, all tables that have been included in the study do not have 'n' specified in the heading and the numbers in the cells don't add up to 3130. Kindly clarify. (Table 1-4)

**Response:** We appreciate the feedback from the reviewer. As suggested, we have specified the ‘n’ in each heading of the table. Since, the data had missing variables the it was analysed separately for every variable. Therefore, the total sample size does not match the ‘n’ for all the variables included in the study.

5. This study is very extensive and has collected a lot of data. However, I think there is too much data for one paper. The emphasis should be on vaccine naiive individuals with few causes of hesitancy being studied.

**Response:** As suggested in the following comment, we have deleted table 7 and 8 to shorten the paper so that it contains valuable data. Table 2 has been added as a supplementary material.

6. Patients who were vaccinated with the COVID vaccine before and questions about cost and effectiveness are best left to another paper. Table 2, 7 and table 4 do not contribute valuable data.

**Response:** As suggested in the following comment, we have deleted table 7 and 8 to shorten the paper so that it contains valuable data. Table 2 has been added as a supplementary material. Table 4 talks about the vaccine acceptance and hesitancy hence we have not deleted the table.

7. The numbers in Table 5a do not add up to 3130. Table 5 b can be added to supplementary data or summarized into one line at the end.

**Response:** Since, the data had missing variables the it was analysed separately for every variable. As suggested, we have added the Table5b to the supplementary data.

8. This paper has a good concept but has too much data. Was a focus group discussion held prior to the study to determine the main causes of vaccine hesitancy? That would help guide us better on what variables to concentrate on.

**Response:** We thank the reviewer for the feedback. But we did not hold a focus group discussion prior to the study. Only open-ended questions were asked to determine the main causes of vaccine hesitancy among the population.

9. Discussion is too long. Kindly shorten it to be shorter and clearer.

**Response:** As suggested, we have shortened the discussion. Additionally, we have divided the discussion into conclusion as well to clearly summarize the key message.

**Reviewer #2:**

1) Fragmentation of sentence needed in line 74-75.

**Response:** As suggested, we have fragmented the sentence as below:

**“***Concerns about vaccine hesitancy are growing globally, especially in populous countries with low literacy. This prompted the WHO to declare it among the top ten health threats in 2019.”*

2) Lines 86-93 are misplaced. It would be wise to elaborate on it in the "Discussion".

**Response:** We appreciate the reviewer’s comment. But, the lines were added to state the prevalence of the vaccine hesitancy rates across the world so as it give an overview of the studies conducted in this domain. Besides, we have mentioned and elaborated the prevalence of the vaccine hesitancy in the “Discussion” as well.

3) Line 99-100- Check for grammar.

**Response:** As suggested by the reviewer, we have corrected the lines to:

**“***The emergence of contagious and/or virulent variants, lower immunity of the vulnerable individuals, and lack of participation in COVID-19 preventive measures may have contributed to a resurgence of the COVID-19 cases*”.

4) Lines 130-152 are misplaced and irrelevant.

**Response:** We thank the reviewer for the comment. The lines were added to provide a brief background of the COVID-19 infection and the vaccination rates in the state to strengthen the rationale behind conducting the cross-sectional study.

5) Choose only one format to discuss the data in lines 298-304. (e.g.- either "10%" or "ten percent")

**Response:** As suggested we have edited the format of the data to maintain the consistency.

6) Repetitive use of words that convey the same message ("efficacy" and "effectiveness") in lines 299-301 should be avoided. Kindly re-phrase the sentence.

**Response:** As suggested the lines have been re-phrased.
