# Supplementary material for: A cross sectional study to examine factors influencing COVID-19 vaccine acceptance, hesitancy and refusal in urban and rural settings in Tamil Nadu, India
Source: PLoS One. 2022 Jun 9;17(6):e0269299. doi: 10.1371/journal.pone.0269299 (PMC9182563; doi:10.1371/journal.pone.0269299)
Supplement: Attachment — Submitted filename: Response to Reviewers.docx [file pone.0269299.s007.docx]

**Response to Reviewers**

Reviewer #1: Thank you for the changes that have been made.
I still feel the article is long and certain points are being repeated in the results section and discussion section.

1. Line 382-384 and 399-402 can be removed. This has already been explained in the comments section.

**Response**: We have deleted the suggested lines.

2. Line 494 comments on a 'third' pandemic wave. Consider changing it to fourth, since we are at the tail end of the third wave in India.

**Response:** Thank you for the feedback. We can changed the third wave to fourth COVID-19 pandemic wave.

3. To make the article comprehensive and easier to read, I would suggest shortening the text explanation before each table. The same information is again listed out in the discussion section.

**Response:** We thank the reviewer for the feedback. We have shorten the text explanation before each table and deleted the few lines listed again in the discussion. We have listed only the key findings in the text explanation before each table.

4. Ethical clearance has been obtained from the institution. However, urban and rural PHC's are not run by the institution (Panimalar) but are run by the State Government of Tamil Nadu. Would a separate ethical clearance be required from the State government to utilize data of patients attending their PHC's?

**Response:** The Institutional Ethics Committee of Panimalar Medical College Hospital & Research Institute (PMCHRI-IHEC) has been approved and recognized by the CDSCO, NECRBHR, Department of Health Research, Government of India. The PMCHRI-IHEC is an independent authority to approve the clinical trials or any protocol involving the human subjects as study participants. Once the protocol is approved by the IHEC, the study can be carried out as per the approved protocol without any deviation across the state as mentioned in the study protocol and as approved by the IHEC. The letter stating the same has been uploaded in the supporting document.

Reviewer #2: The manuscript is still a bit lengthy and sometimes wavers from the topic. Lines 110-120 in Introduction and lines 144-155 in Materials and Methods may be removed/cut-short as they seem inappropriately placed and are making the manuscript unnecessarily lengthy.

**Response:** Thank you for the feedback. We have removed the suggested lines (110-120) to make the article comprehensive. We have cut-short the lines 144-155. We have also deleted the lines 133-137 to shorten the manuscript.
